# Researches, Physiological and Anatomical

**Published:** 1841-07

**Authors:** 


### Art. VIII.

*Researches, Physiological and Anatomical.* By John Davy, M.D. F.R.S. In Two Volumes; with Plates.—*London,* 1839. 8vo, pp. 455-80.

The style in which these elegant volumes are presented to the public is perhaps alone sufficient to ensure for them a favorable reception, independent of the reputation of their author, and the fact that they contain the whole of his scientific memoirs. But the former of these recommendations is trifling in comparison with the real value of their contents, which include some of the best physiological papers in our language. In collecting these together, from the transactions of the different societies and scientific journals through which they have remained scattered for a series of years, and in now presenting them in an amended form, we feel that Dr. Davy has rendered good service to the public; since, however valuable may be the labours of even the most distinguished observer, they often remain almost inaccessible to the generality of enquirers, when published only in the expensive and ponderous volumes of scientific bodies, which are rarely to be found except in the libraries of the most affluent and of public institutions, and not always in them, as we have frequently had cause to experience.

The *experiments and observations on the Torpedo,* which form the subject of the first paper, and which were communicated to the Royal Society in 1832 and 1834, and subsequently published in the Transactions, are equally interesting to the physiologist and comparative anatomist. Up to the period of these observations "it had not been ascertained," as Dr. Davy remarks, "that the electricity of the torpedo, considering its peculiar influence to be electrical, had either the power of acting electro-chemically, in separating the elements of any compound bodies, or magnetically, either in affecting the needle in the multiplier, or in imparting magnetism to iron; or lastly, of generating or producing heat: points to which my experiments were particularly directed, and with positive and successful results." (p. 3.) The ascertainment of these facts was an important advancement in our knowledge of the phenomena of animal electricity; and, as it appears from the remarks of Dr. Davy, had been looked forward to by his brother Sir Humphry as likely to prove conclusive as to the nature of that astonishing power, whether, as he imagined, it be of a peculiar kind, or, as formerly believed by Mr. Cavendish and recently taught by Dr. Faraday, it is merely another form of common electricity. Upon this question Dr. Davy does not give a decided opinion, but points out distinctly the circumstances in which the electricity of the torpedo resembles the known kinds of electricity, and also those in which it differs from them. Thus it resembles common and voltaic electricity in magnetising and polarizing iron, and in producing chemical changes; but it appears to differ from common electricity in affecting the multiplier and producing these changes more distinctly, while its power of passing through air, and also of producing heat, is considerably less. On the other hand, as compared with voltaic electricity, it acts more feebly on the multiplier, and decomposes water and metallic solutions less readily, but the shock produced by it and its power of magnetising iron are greater. In addition to these, Dr. Davy remarks, "there are other points of difference, . . . in which the metallic communication was interrupted by a strong solution of salt. In this instance the full power of the fish appeared to pass; water was decomposed, a shock was received, needles were magnetised, and the multiplier was affected. When the same experiment was made on the electricity excited by the small voltaic combination of a single plate of copper and zinc, each less than an inch in length and half an inch in breadth, immersed in an acid, neither water was decomposed nor was the multiplier affected. When it was made on the electricity of the electrical machine by means of a Leyden jar, all the effects were witnessed excepting the motion of the multiplier, and the order of succession of poles in the needles magnetised in the spirals." (p. 45.) Dr. Davy very naturally enquires how these facts are to be explained, but admits that the majority of them are in support of the opinion that animal electricity is identical with the other known forms of this peculiar and astonishing agent.

These experiments naturally lead to a closer examination of the electrical organs themselves, both in regard to the structure of the parts which appear to be most concerned in producing the effects, and also in regard to the differences which these parts exhibit in their formation in the different kinds of electrical fishes; and further also, in what respect these parts differ from the corresponding ones, if any exist, in species closely allied to the electrical fishes, but which have never yet been known to evince any electrical phenomena.

The first portion of this enquiry, the structure of the electrical organs, like almost every other subject connected with animal life, was long ago undertaken by Hunter, and in repeating his dissections, Dr. Davy found that his own observations very closely agreed with those of our great anatomist. Hunter described the electrical organs however as abundantly supplied with blood, but Dr. Davy did not find this to be the case in the recent fish, since the blood-vessels themselves are small, and the quantity of blood sent to the organs is inconsiderable. In accordance with this observation he found also that the specific gravity of the electrical organs, as compared with other parts of the body, is remarkably low. Thus the organs of a full-grown fish had a specific gravity of only 1·026, as compared with water at 1000, while the specific gravity of the abdominal muscles of the same fish was 1·058, and of the dorsal of 1·065. He remarks, however, that the integuments and mucous glands which surround the organs are exceedingly vascular, a circumstance which probably led Hunter to regard the organs as well supplied with blood. From a consideration of all the facts, Dr. Davy thinks it "very difficult to resist the conclusion that the electrical organs of the torpedo are not muscular, but columns formed of tendinous or nervous fibres, distended by thin gelatinous fluid. Their situation too, surrounded by and exposed to the pressure of powerful muscles, shows that if condensation is required for the exercise of the electrical function, they may experience it without possessing any muscular fibres of their own substance. The arrangement of the muscles of the back and of the fins, and of the very powerful cross muscles situated between the under surfaces of the electrical organs, are admirably adapted to compress them." (pp. 33-4.) For this purpose Dr. Davy thinks they are designed.

The nerves supplied to these organs are acknowledged by all anatomists to be exceedingly large. They consist of three distinct pairs, derived in the torpedo entirely from the cerebral structures. This fact deserves especial notice, since this is not found to be the case in the other electrical fishes, in each of which, as we shall find, the nerves are derived from different sources. Hunter had accurately described the origins and trunks of the electric nerves in the torpedo, and Dr. Davy has followed up the investigation of their minute distribution with great care. The description which he gives very closely agrees with that given by Mr. Swan, of corresponding nerves in the common skate, (*raia batis*,) a fish nearly allied, in general structure and habits, to the torpedo. But Dr. Davy has not so accurately identified the individual nerves in the torpedo as has been done by Mr. Swan in the skate. He seems to regard what we believe to be in reality divisions of the fifth and vagus, as *peculiar* electrical nerves; and remarks,—" It is an interesting fact that the gastric nerves are derived from them." (p. 37.) A reference to Mr. Swan's excellent delineation of the nerves of the skate * will materially assist us in identifying those of the electric organs in the torpedo. On comparing the description given of these by Dr. Davy with the description and delineation of those of the skate by Mr. Swan, we entirely agree with Desmoulins and Majendie,† that of the three great trunks which go to the electric organs, the two anterior ones are branches of the third division of the fifth, and the third, or posterior one, of the vagus.

In regard to the connexion of the electrical apparatus with the processes of nutrition, it is remarked by Dr. Davy that,—" In the instance of a fish which I had in my possession alive for many days, and which was frequently excited to give shocks, digestion appeared to have been completely arrested; when it died, a small fish was found in its stomach, much in the same state as when it was swallowed; no portion of it had been dissolved." But although in this instance the function of digestion appears to have been suspended, we cannot agree with him that only " *superfluous* electricity, *when not required for the defence of the animal*," may be directed to this organ to promote digestion. (p. 37.) We should rather ascribe the circumstance above noticed simply to the known principles of animal life, in accordance with which it is found that when the energy of one set of organs is more than usually excited, that of all others is greatly interfered with; as for instance, when the function of the brain is much disturbed in the human subject, digestion, as is well known, is impeded, and often becomes entirely deranged, whether the exhaustion of nervous power be the result of disease, of mental emotions, of violent muscular exertion, or of the stimulus of over-excitement of the generative system. The exhaustion of nervous power in giving shocks was doubtless the cause of the circumstance noticed in the torpedo, the arrested digestion being the result of deficient energy in the whole system, occasioned by over-excitement of one set of organs. But a further observation by Dr. Davy points more directly to the use of the electrical function, as connected with nutrition, a view supported not only by the power which the electrical fishes are now known to possess of decomposing compound bodies, but also by the peculiar habits of the fishes themselves, which, although belonging to different genera, re-

* Illustrations of Comp. Anat. Nervous System. (p. 2, pl. x.)

† Anatomie Compar. des Syst. Nerv.

side either at the bottom of deep and muddy waters, or in dark and shaded places, and but seldom approach the surface. On this Dr. Davy remarks, "It seems probable that as the branchiæ are liberally supplied with twigs of the electrical nerves, there may be some connexion between its respiratory and electrical functions; and I venture to offer the conjecture, that by means of its electricity it may have the power of decomposing water and of supplying itself with air when lying covered with mud or sand, in situations in which it is easy to conceive pure air may be deficient; and in my experiments I have often fancied that I have witnessed something of the kind; after repeated discharges of its electricity, the margin of the pectoral fins has acquired an appearance as if very minute bubbles of air were generated in it and confined." If it be hereafter found that the whole of the electrical fishes possess the same property, the chief use of the organs, and their connexion with the function of nutrition will then be sufficiently indicated; and it will also be evident why the electrical power is so directly under the control of the will of the animal, as it is proved to be by the experiments of Humboldt and others. Humboldt divided a gymnotus in the middle of the body, and found that shocks were given only by the anterior half, which retained its connexion with the brain, although the electrical organs in the anterior as well as the posterior half are supplied with nerves only from the spinal cord. In addition to these circumstances, that of the electrical function being possessed by the torpedo, even in the fœtal state, as found by Dr. Davy, long before it could have any necessity for exercising this function as a means of defence, seems further to indicate that its primary use has reference rather to some organic function connected with the growth of the animal itself, than to its mere defence; and this is still further supported by the fact that the electrical power is more energetic in the young than the adult state.

We must not leave this part of our subject without expressing a hope that opportunities may hereafter be afforded to Dr. Davy of prosecuting similar enquiries on the anatomy and physiology of the other electrical fishes, and thus further assist in elucidating the nature of this most mysterious of all the properties of animal life.

Before passing to the strictly physiological papers in these interesting volumes, we shall briefly examine the most important of those which are chiefly anatomical. The first of these, on the *urinary organs of the amphibia*, is of considerable interest to the comparative anatomist, from the circumstance that the correct anatomy of these parts is yet a matter in dispute. This difference of opinion, which has evidently arisen from insufficient comparative examinations of the urinary organs, not only of the true amphibia, but also the neighbouring classes, the observations of Dr. Davy will probably tend to reconcile. Thus it is disputed whether the true amphibia, the frogs, toads, and salamanders, do really possess a urinary bladder; the vesicle, which in those animals contains a fluid, and is attached to the intestine, being regarded by some as the proper urinary organ, and by others as simply a membranous receptacle or water-bag, in which fluid is collected, as in a reservoir, to supply the aqueous expenditure from the surface of the body of the animal. That it is a true urinary bladder appears to be the opinion of Cuvier, Grant, and many others; while Townson, Dumeril, Altena, and Thomas Bell, believe that it is simply a membranous receptacle, for the purpose just stated, and that the true urinary bladder is absent. From the observations of Dr. Davy and ourselves on the true amphibia, and of Dr. Davy on the chelonian, saurian, and ophidian reptiles, we are led to the conclusion that the vesicle in question is indeed the true urinary bladder, notwithstanding that the ureters do not terminate in it.

The biauricular structure of the heart in batrachian reptiles, which constitute the subject of the next anatomical paper, was first noticed by Dr. Davy in 1824, but seems to have excited little attention, until the publication of Professor Owen's interesting paper on the same subject in 1834. It is remarkable, however, that even this important fact which, although it escaped the observation of all other anatomists, had previously been observed by our great countryman Hunter, as is proved by a preserved copy of some of his destroyed manuscripts, and had actually been made part of the basis of a classification by him, although the fact itself was even overlooked by Cuvier, and not again noticed by any other anatomist until rediscovered by Dr. Davy. We may refer to this circumstance as a still further proof, if any were wanting, of the necessity for a careful revision and republication of scientific papers during the lifetime of their author. Had this been done with the papers and manuscripts of Hunter, there can scarcely exist a doubt that many valuable facts that are now lost to science would have been preserved, and perhaps have been the foundation of important discoveries, which may yet be many ages before they are brought to light. And many, too, of the most captious enquirers, both in this country and on the continent, who now lay claim to the honour of originality, would have found their labours to a great extent anticipated by those of this giant in anatomical knowledge.

The next paper we shall notice is on *the structure of the ductus communis choledochus, and on the flow of the bile.* In this paper are described some peculiarities of structure which have hitherto escaped the observation of anatomists, even of those who have paid most attention to the structure of the liver and its appendages. We have looked for some notice of this structure in Mr. Kiernan's valuable paper on the liver, but have not been able to discover any indication of his being acquainted with it. Dr. Davy states that after emptying the tube, and washing away the adhering mucus, "the lower part of the ductus communis choledochus and of the pancreatic duct, will be seen provided with a kind of valvular apparatus, composed of delicate angular processes or projections of their inner coat, pointing downwards." (Vol. ii. p. 427.) This structure is sometimes more, and sometimes less developed.

"The processes or valvular projections commonly first make their appearance (tracing the passage downwards from the liver) a little above the place of junction of the pancreatic duct, and are continued to the termination of the common duct in the intestine. In the pancreatic duct they are more limited ; they are confined to its mouth, and occasionally to a small portion of the tube adjoining, not more than two lines from its mouth. Their valvular nature is easily shown by the introduction of a probe, directed downwards, towards the intestine, it passes smoothly without obstruction; but in the contrary direction, towards the liver, it is impeded and stopped, arrested by the minute sacculi, which exist at the base of the processes. The use of these processes is implied in their valvular nature and situation: whilst they do not stop the descent of the secreted fluids, which it is the office of the ducts to discharge; they are well adapted to prevent the pancreatic juice from ascending either into the gall-bladder or the liver, and the bile from flowing or penetrating into the pancreas." (Vol. ii. p. 428.)

This structure had not been noticed even by comparative anatomists, but it has been found by Dr. Davy, on extending his enquiries to the domestic animals, to exist in most of them, in which it closely resembles the corresponding part in the human subject.

The next paper which we feel ourselves called upon to notice is one of considerable pathological interest. It is on the *closure of the chief arteries rising from the arch of the aorta.* It contains the details of a case in which the principal arteries which proceed from the arch of the aorta were gradually closed, without loss of life, and continued so for nearly two years, as was indicated by the entire absence of pulsation in the upper extremities and neck. The death of the patient took place in consequence of a rupture of the aorta near its base, the aorta itself being the seat of an aneurism six inches in height and four in width, which Dr. Davy believes to have been of slow formation. From this case, compared with that of another of aneurism of the same parts, but in which, instead of the dense fibrous coagulum which nearly filled up the cavity of the first, leaving only a passage for the blood on the inferior or concave side of the arch, the inner coat of the artery was thickened, and contained thin plates of bone and depositions of atheromatous matter; Dr. Davy concludes that the great arteries of the upper parts of the body may be tied in operations on the human subject without loss of life. The practicability of this in the rabbit and dog has already been demonstrated by Sir Astley Cooper in the first volume of Guy's Hospital Reports. From a comparison of these cases, Dr. Davy states his belief that the closure of arteries takes place much more frequently than is generally supposed, even in a state of general health.

The next paper, on the *rupture of the heart and aorta,* is of consequence, chiefly from the rarity of well-authenticated cases of this accident; but for its details we must refer to the work itself.

Another of Dr. Davy's anatomical memoirs relates to the *specific gravity of different parts of the body.* The application of specific gravity as a means of detecting disease will probably be of most use with reference to change of structure in the viscera, but although at present it can only be employed as an imperfect means of diagnosis, it may ultimately prove, in the hands of a Laennec or an Andral, highly important, when a sufficient number of careful observations has been made upon which to establish exact general conclusions; since, as Dr. Davy justly remarks, there is probably no organic change in the structure of any part of the body which is not accompanied by a corresponding change in its specific gravity.

The paper *on the age of morbid adhesions and fluid in the pericardium* is of much pathological interest. Dr. Davy remarks that it is a pretty generally received opinion, that the age of these formations may be nearly guessed at by their comparative degrees of strength; but experiments have led him to believe that this opinion is not correct, since "wounds, it is well known, which heal by the *first intention* are often firmly united in twenty-four hours;" and he remarks that, " in the same space of time, when inflammation has been artificially excited, he has witnessed strong adhesions."

This rapid formation of adhesions the author refers to the coagulated lymph effused from the wound, which quickly becomes "*first viscid*, and afterwards solid." While viscid, and when still transparent, " it has the tenacity of mucus, and admits of being drawn out into fibres and bands, which soon becoming firm and opaque, very well represent the ordinary adhesions of the lungs, and in a very few hours attain their maximum of strength." It is entirely upon this *viscidity* of coagulable lymph, that Dr. Davy believes the formation of these adhesions to depend; a property of lymph which, he remarks, has not before been noticed by authors, who usually explain the occurrence of adhesions without reference to this peculiarity. On the above experiment Dr. Davy founds his opinions, and we think justly, that the age of morbid adhesions cannot always be correctly estimated by their comparative strength.

Respecting the effusion of fluids into the serous cavities, he is again opposed to the commonly received opinion, in believing that it takes place only during the life of the animal. But although the experiments on which this belief is founded are of great value, we do not think they are quite conclusive, inasmuch as they were made only on perfectly healthy animals, and not on those suffering from disease of the parts in which the effusion was expected to take place. Now we are free to admit that effusion may not take place after death in parts which during life are perfectly healthy, but we are disposed to think that, in those which have becomed diseased, and in which only effusion is usually found, when this has commenced during life it may continue for a short period after death. This view is in accordance with the already well-known permeability of animal membranes, and also with a fact noticed by Dr. Davy, that in proportion to the tendency to disorganization in a membrane, such is the facility with which it allows fluids to pass through it.

The *observations on pus* are of a miscellaneous description, but, like the subject last noticed, are of considerable pathological interest. They consist of several distinct enquiries concerning the nature and peculiarities of this fluid. The first of these was to ascertain whether pus contains any admixture of atmospheric air. All the experiments made showed a negative result. The second enquiry was to examine the specific gravity of this fluid, which has usually been stated to be 1030. In this investigation Dr. Davy found as might, *a priori*, have been expected, that the specific gravity of this fluid is variable in different instances. In seven distinct examinations it was found in one so low as 1028, while in another it amounted to 1042. This great difference is attributable to two circumstances, the proportions of the different ingredients, and the variable density of the liquid part. The pus-globules, he believes, are of not less density than the blood-corpuscles, an opinion to which he has been led by direct experiment. In other experiments Dr. Davy found that pus freezes less rapidly than water, and that when thawed it does not regain its original properties. From his experiments on the composition of pus, he has satisfied himself that the analysis of this fluid recently given by Dr. Gueterbock* is correct, and deserving of confidence, it

* De Pure et Granulatione, p. 17; Berol., 1837.

being confirmed generally by the results of his own investigations, although he has followed in these a method of analysis different from that author. Dr. Gueterbock's analysis is as follows:

| | |
|---|---|
| Water . . . . . . . . . | 86·1 |
| Fatty matter soluble only in hot alcohol . . . . . | 1·6 |
| Substances soluble in cold alcohol, viz. fatty matter and osmazome | 4·3 |
| Substances soluble neither in hot nor cold alcohol, viz. albumen pyina, and the substances of the globules ("globuli et grana puris") . . . . . . . | 7·4 |
| Loss . . . . . . . . . | ·6 |
| | 100·0 |

We cannot afford space for an examination of all the remaining papers on detached subjects in these interesting volumes, although many of them are of great value, particularly those on the action of corrosive sublimate, and on that of lime and of tannin on animal and vegetable substances, but shall content ourselves with briefly noticing those on the poisons of some reptiles,—the toad and snakes of Ceylon,—before passing to the consideration of others which have constituted Dr. Davy's favorite enquiries, the temperature of animals and its connexion with the functions of circulation and respiration.

It has long been a popular opinion that the toad secretes a peculiar poison, and dogs have often been said to have suffered from affections of the mouth and fauces after having bitten this reptile. This opinion has been almost universally rejected by naturalists; but Dr. Davy states that his own investigations have led him to the conclusion that the vulgar notion is not without foundation, and that a peculiar fluid is contained in follicles of the skin, more especially about the shoulders and neck of the animal, which he has collected from old and large animals in a quantity sufficient for the purposes of examination. This fluid, he states, is of a more acrid nature than the poison of most venomous snakes, but is less fatal when absorbed into the circulation, and hence he conceives it to be analogous rather to the fluid secreted by the bee and scorpion than to that of the poison of snakes. We cannot subscribe to this opinion. The poison of the insects named is secreted by distinct glands within the body, no way connected with the tegumentary structures, and in our opinion is more analogous to that of the snake, which like it is secreted within the body, than to the fluid from the cutaneous follicles of the toad. The poison of the bee certainly appears to act more locally when absorbed into the system, than that of the snake, at least in the higher vertebrata; but this is not the case in its effects on the invertebrata; to them it is a most deadly poison and is followed by fatal results when injected into the body in a very few minutes, as every one is convinced who has witnessed its effects during the combats which are so well known to take place in the hive. Neither are its effects to be despised when injected even into the human body in sufficient quantity, as many fatal cases are known to have resulted from its introduction.

Dr. Davy seems to think that the poison of the toad may in some way be connected with the function of the skin and perform a part in the economy of the animal; which certainly is not the case with that of the insect, which must be regarded as an offensive and excretory fluid. He conceives that "it may serve to separate a portion of carbon from the blood, and thus in its function be auxiliary to the function of the lungs." In support of which notion he remarks, "I have found the pulmonary arteries of the toad, each divided into two branches, one of which went to its respective lung, and the other very little smaller, to the cutis between the head and shoulders on each side, and was extensively ramified where the largest follicles were situated, and where there was a plexus of veins of great size, as if intended for a reservoir of blood." (p. 111.) His reasoning, however, appears to us inconclusive: as it is well known that the skin of the batrachia is an important organ of atmospheric respiration, between which and the secretion of poison there does not seem to us to be any connexion.

The poison of animals is a subject that well deserves the closest attention, since its *modus operandi* in most cases is at present involved in the greatest obscurity. At one time its introduction into the living body is immediately followed by the most violent and fatal results, and at another by scarcely any apparent effect, even when the injury in both cases has been inflicted by the same animal. This is fully shown in the account given by Dr. Davy of his experiments on the *poisonous snakes of Ceylon.* These were made to ascertain what are the effects of their poison, and were instituted to smooth the way to determine, if possible, the action of each poison, and furnish data for inferring what are the pure effects of the poison, what of the powers of nature opposing its effects, and what those of the medicines administered. In no subject is discrimination more required than in this mysterious one; and in no one perhaps has less judicious discrimination been used. It has often been taken for granted that the poison of all snakes is similar,—not differing in its kind, but only in its intensity of action; and, agreeably to this assumption, that the medicine useful in one instance must be serviceable in all. And too often, medicines have got into repute as antidotes from being given in slight cases, in which recovery would have taken place without medical treatment,—beneficial changes that were due merely to the preservative powers of the constitution. (Vol. i. p. 132.)

We fully coincide with these observations, which are amply exemplified in the experiments on the snakes of Ceylon. The subject of these experiments were the hooded snake, *naia tripudians;* the carawilla, *trigonocephalus hypnale;* and the tic-polonga, *vipera elegans.* Of these the latter, like the European species of the same genus, is the most venomous, and its bite is usually fatal; that of the hooded snake is fatal in a less degree, and the carawilla but very rarely so. All the experiments showed that the direct effects of the poison of each species were very different. The details of them possess much interest, but they are too long for quotation, and would not well bear abridgment; so that we must refer those interested in the subject to the volumes themselves.

One remarkable circumstance connected with the poisoning of these reptiles is that the most venomous appear to occasion the slightest local inconvenience at the moment of the introduction of the poison, the subject bitten being often unconscious that he has been wounded. This was noticed by Dr. Davy in his experiments on fowls, and was also noticed in a case of a man bitten by a rattle-snake. In that instance no pain whatever was felt by the wounded party on the introduction of the poison; the effects of which, when they became apparent, were at first trifling and local, although followed by a fatal result in less than twelve hours. Notwithstanding the rapidity with which death takes place in some instances of the bite of venomous reptiles, there are good reasons for believing that in every instance the poison has already entered the general circulation before any ill effects are produced.

On the medical treatment of these injuries Dr. Davy offers no opinion, but merely remarks that oil, both when administered internally and when applied externally, has been supposed to do good in cases of poisoning by the viper, and that arsenic has seemed to do good in some instances of poisoning by the hooded snake. It is probable that there is no specific remedy for any of these injuries, but that, as Dr. Davy suggests, the bite of each species of these reptiles may require a different mode of treatment according to the nature of the poison. The first and most important indication in every instance is to excise the bitten part, and by the application of cupping-glasses and ligatures to prevent as much as possible the poison from being absorbed into the circulation.

In examining the details of these experiments we have been struck with the great similarity of the effects produced by the poison of the tic polonga to those of poisoning by prussic acid. Thus both destroy life with great rapidity, both quickly deprive the muscular fibre of its power of contraction, and both, under certain circumstances, prevent the blood from coagulating. But it is worthy of remark that in this latter respect the two poisons appear to differ or to resemble each other according to the rapidity or the slowness with which they occasion death. Thus when a fatal result is produced by the poison of the snake within a few minutes, the blood is instantly coagulated both in the arteries and veins; but if death be not occasioned quickly, and an hour or more elapse, then the blood remains fluid in the vessels, as in the case of poisoning by prussic acid. This similarity in the principal effects of these poisons has induced us to believe that the employment of powerful diffusible stimuli, as ammonia, &c. both internally and externally, may be attended with benefit in cases of poisoning by the tic polonga, as it is believed to be in cases of poisoning by prussic acid, and by the common viper, a reptile far less deadly than the tic, but which seems to produce the same disposition in the blood to coagulate and become congested in the vessels; as is evidenced by the well-known swelling, lividity, and pain which quickly follow the introduction of its poison.

Before closing these remarks we cannot refrain from noticing a circumstance which fell under our own observation in respect to the slow effects of prussic acid on the common snake. We were endeavouring to kill a full-grown specimen for the purposes of dissection, by pouring into its mouth about a drachm of Scheele's medicinal prussic acid. After waiting a few minutes for the result we were astonished at finding that the poison had produced no marked effect on the reptile, which still continued very lively. A second dose of more than double the same quantity was again poured into its mouth, but it was not until after several minutes had elapsed that the poison seemed to begin to exert its deadly influence. A friend has mentioned to us an occurrence of the same kind in regard to the turtle. The explanation is probably to be found in those peculiarities of constitution in reptiles which confer upon them such remarkable power of enduring severe injuries.

The various papers on animal heat contained in these volumes include some of the most interesting portions of Dr. Davy's labours, and have added greatly to our knowledge of this important subject, of which Dr. Davy has proved himself one of the most careful and accurate investigators. His success in these enquiries has evidently depended greatly upon his having followed in all his experiments that mode of proceeding which we are glad to know is now more generally adopted than formerly, and which it is to be regretted has not always been pursued by physiologists: the carefully collecting of a large number of facts at the outset of each investigation, upon which to base his opinions, before any fixed theoretical notions had taken forcible possession of the mind of the investigator, a course which alone enables the enquirer to deduce correct and impartial conclusions. The importance of this method is fully estimated by all who have had the patience to pursue it, as being that alone which will lead to the establishment of a solid reputation, the foundation of which is not to be shaken, however much assailed by plausible criticism or brilliant theories. In pursuing this method Dr. Davy has succeeded in establishing many important truths.

In the *account of some experiments on animal heat*, one of the earliest and most valuable of these papers, are announced the important fact of a difference of temperature between the two sides of the heart, and also between venous and arterial blood. Dr. Crawford had assumed, as the basis of his theory of animal heat, that there is a difference in the capacities of the two kinds of blood for caloric, and an uniformity of temperature of the blood in the two ventricles; the heat of the body, according to his views, being supplied by the arterial blood, which he supposed contained more latent heat than the venous, and which it derived directly from the air during respiration, and liberated again during the conversion of arterial into venous blood. The facts ascertained by Dr. Davy are strongly opposed to this theory. Besides an absolute difference in the temperature of venous and arterial blood appreciable by the thermometer, he found that there is in reality scarcely any difference in the capacities of the two kinds of blood for heat in the latent form. In his most carefully performed experiments on the blood of the sheep, he ascertained that the specific gravity of venous blood was 1051, and its capacity for heat 903, while that of arterial was 1049, and its capacity for heat 913, a difference so trifling as to be totally insufficient for the support of Crawford's theory. These were the numbers considered by Davy as the nearest approximation to the truth, but we are nevertheless inclined to suggest whether there may not be constantly some variation in this respect in venous and arterial blood at different ages, and perhaps also at different seasons of the year; since in another set of experiments on the blood of a lamb four months old, the relative capacities appeared to be as 922 for the venous, and 934 for the arterial blood; and in a third set on a lamb five months old, the specific gravity of venous blood was 1050, and its capacity for heat 852; while the specific gravity of arterial blood was 1049, and its capacity for heat 839. The difference of temperature in the blood of the two sides of the heart has not been observed by all experimenters. Krimer, Scudamore, and Meyer have noticed the difference of temperature in venous and arterial blood, but the latter author could not observe that of the two sides of the heart. We are ourselves satisfied of the general correctness of Dr. Davy's observations on this subject, but do not think that this difference is so great as has usually been stated. Meyer noticed a difference of from 2¼ to 4½ degrees of Fahrenheit between the blood of the jugular vein and that of the carotid, but yet failed to observe a difference between the two sides of the heart. This circumstance leads us to suspect that there must have been some error in performing the experiment. The exposure of the vein for a few seconds, before ascertaining the temperature of the blood within it, would sufficiently account for the excess of difference between that and the blood of the carotid. We believe Dr. Davy's statement of the amount of this difference is much nearer the truth. The failure of many observers to notice a difference of temperature in the right and left ventricle may perhaps have arisen from a circumstance noticed by Davy in explanation of the discrepancy between his own observations and those of Messrs. Coleman and Cooper—namely, that when the animal has been killed by asphyxia, and there is an accumulation of blood in the right ventricle, he has then observed but little difference in the temperature of the two sides of the heart: a circumstance noticed also by ourselves, and which he explains by supposing that in all cases of impeded respiration the blood returning from the lungs to the left ventricle having been insufficiently or not at all acted upon by the air, little or no development of heat has taken place, while there is usually an accumulation of venous blood in the right ventricle, and the temperature in the two cavities is in consequence nearly similar. We are not so well satisfied with the details of the experiments on the temperature of other parts of the body as with those on the heart, since they appear to savour a little of preconceived notions. The great objection is the length of time that elapsed in making them, which occupied about an hour, during which the body was exposed; and, as the notice of the slight degree of pyrexia which followed evinces, together with the quickened pulse, unpleasant sensation of chilliness and parched mouth, clearly prove the body suffered from its unusual exposure to a detrimental influence. Of the fact of a general difference of temperature of different parts of the body we have not the slightest doubt, both as regards the external parts and the internal organs, although this difference may not be so great as has sometimes been supposed, nor as Dr. Davy's experiment on the temperature of his own body seems to indicate. Experiments of this description, in order to approach to the truth, ought to be made under circumstances as nearly as possible similar to those in which the body is usually placed, which was not the case in the experiment now alluded to. One of the most remarkable circumstances mentioned by Dr. Davy is that respecting the temperature of the brain, as compared with the temperature of *the rectum*. He had noticed this circumstance in some of his earliest and most carefully performed experiments, and, as he well remarks, surprised at the fact, was led to repeat his experiments upon four lambs. The temperature of the air at the time being 68°

Fahrenheit, the mean of the four observations on the brain was 105·18° Fahrenheit, and that of the rectum in the same animals 105·81° Fahrenheit, thus indicating an excess of temperature in the rectum of 0·63d of a degree of Fahrenheit; and apparently confirming the opinion that the source of animal heat is directly connected with the function of the lungs. In one only of these experiments was there any excess of temperature in the brain over that of the rectum, and that excess amounted only to 0·25th of a degree, while the maximum of temperature in the rectum, in one instance, was 1·75° Fahrenheit. Notwithstanding these apparently conclusive results, the fact was questioned by Professor Müller; and Dr. Davy in consequence was led to repeat his experiments in December and January, 1837-38, when the temperature of the air was 44° and 45° Fahrenheit, and again obtained similar results, but with much less difference than in his former experiments. Thus in eight observations, there were two in which the temperature of the brain exceeded that of the rectum, each one degree Fahrenheit; and one in which it was of exactly the same temperature, while in one instance only did the maximum of excess in the rectum amount to so much as one degree. But Dr. Davy considers these experiments as generally confirmatory of his preceding ones. On examining the mean of these experiments we find it is exceedingly trifling, and amounts only to 0·13th of a degree Fahrenheit, which, connected with some observations we ourselves have made on this subject, leads us most decidedly to agree in opinion with Professor Müller, that there has been some error of observation. Nevertheless we agree with Dr. Davy, that this apparent difference marks in a decided manner the effect of cooling influences on the brain; and we are fully satisfied that, "excepting the hands and feet, the brain appears to lose its heat more rapidly than any other part of the body," as is shown in his *observations on the temperature of the human body after death*, to which we shall presently refer.

The *observations on the temperature of man and other animals*, first published in 1826, have been constantly referred to as containing, like all Dr. Davy's papers, much valuable information. The first subject examined is the variation of temperature to which the human body is liable in passing from a temperate to a tropical climate, and in descending from a hilly country, where the temperature of the air is moderate, to a low one, where the diurnal variation is very considerable; and next the temperature of different races of men and kinds of animals. On each of these subjects Dr. Davy has succeeded in establishing new facts, which have since been further confirmed by other observers. It is to these observations that we are indebted for our knowledge that the human body acquires an increase of temperature in passing from the temperate to the torrid zone, a circumstance which, although naturally to be expected, was left in doubt by the previous observations of Dr. Franklin on the temperature of his own body. The observations before us were commenced on the 10th of March at noon, in latitude N. 9·42°, when the atmosphere was 78° Fahrenheit. The mean temperature of seven individuals in good health was then 98·85°. We have deduced this from the details given in the paper. On the 21st of the same month, in latitude N. 0·12° at noon, with the sun apparently vertical, the sky clear, a fresh breeze blowing, and the temperature of the air 79·5° Fahrenheit, that of the same individuals was 99·21° Fahrenheit. On the 4th of April, in latitude S. 23·43, at between twelve and one o'clock in the afternoon, the weather very fine and temperature of the atmosphere 80° Fahrenheit, the mean of that of the same individuals was 99·67° Fahrenheit; and lastly, on the 5th of May, in latitude S. 35·22° Fahrenheit, with the weather damp and cool, the temperature of the air only 60° Fahrenheit, and when a general sensation of chilliness was felt, it had sunk to 98·28° Fahrenheit. No continued series of observations have yet been published on the variation which the temperature of the body undergoes in low states of the atmosphere, so that as yet we have but insufficient means of judging of the amount of this variation in a state of good general health. Observations similar to the above were made on the change of temperature experienced in passing from a high mountainous district with a comparatively low temperature of the air to a low one with a high temperature, with the same general results as those above stated. Thus in passing from Kandy, in Ceylon, which is elevated about 1500 feet above the level of the sea, to Trincomalee, which is about fifty miles distant from any mountains, and has a mean annual temperature of from ten to fifteen degrees higher than Kandy, the temperature of the body was increased, and on returning to the same place was again diminished. Thus six individuals before leaving Kandy, when the atmosphere was 69° Fahrenheit, had a mean temperature of 98·15°; and in a few days afterwards at Trincomalee, when the air was 82° Fahrenheit, and when they had been leading a sedentary life, it was increased to 99·9° Fahrenheit, a difference of more than a degree and a half. Thus, although it must be borne in mind that in the latter instances the individuals had breakfasted but two hours previously, a circumstance for which some allowance ought to be made, it is evident that they had acquired an actual increase of temperature, which was still further proved by the fact that on their return to the mountainous district, when the mean temperature of the air was the same as before leaving, that of their bodies had subsided again to about 98° Fahrenheit. That this variation of temperature does not depend on the individuals examined belonging to a different race from ourselves, was proved by observations on many individuals of different races and tribes, both at the Cape of Good Hope and Ceylon, and the Isle of France; on the Hottentot, the Negro, the natives of Madagascar and Mozambique; the Caffres, the Vaidas, Singalese, Malays, Sepoys, and half-caste. In none of these, as Dr. Davy remarks, did the temperature vary more than it is found to do among any similar number of Europeans, and consequently the slight differences observed between them could not be attributed to difference of race or habit.

In the observations on the temperature of the different kinds of animals, we observe the same general accuracy and care as in those on man. We shall briefly notice the most interesting of these, remarking, however, that in a few instances we have reason to believe that the amount recorded is rather below the general temperature of the animals. Thus in the domestic cat in this country, when the temperature of the air in September was 60° Fahrenheit, that of the cat was 101° Fahrenheit; and in Kandy when the air was 79°, that of another specimen was 102° Fahrenheit; now we have observed it in this country in the summer, when the temperature of the atmosphere has ranged between 65° and 70° Fahrenheit, amount to from 102·5° to 103·5° Fahrenheit. Again, the temperature of sheep in Scotland during the summer, is said to vary from 101° to 104° Fahrenheit; at the Cape of Good Hope, in winter, from 103° to 104° Fahrenheit; and at Ceylon, when the air was 78° to 105°. We have ourselves observed a much higher temperature than is here recorded in the sheep of our own country, and that too when the air has been scarcely more than 60° Fahrenheit. The temperature of the common fowl has been given with more correctness. At Edinburgh, in winter, when the air was 40° Fahrenheit, the temperature of a full-grown hen was 108° Fahrenheit; and at Mount Lavinia, in December, when the air was 78° it was found to be 110° Fahrenheit; and in the instance of some chickens in was 111° Fahrenheit. The temperature of the common duck was similar to that of the fowl.

The observations on the different classes of reptiles correspond with those which had previously been made by Martine* and John Hunter;† and also with the more recent and numerous cases of Czermak,‡ Welford,§ and Berthold,|| in so far as that all of these observers have noticed a higher temperature of body than that of the surrounding medium when this has remained stationary, and the animals have been in a state of activity. Hunter's experiments, made as they appear to have been without reference to these circumstances, seem at first somewhat contradictory. Thus in some instances he found a higher, and in others a lower temperature than that of the surrounding medium; but this discrepancy is cleared up by the experiments of others. The cause of the apparent non-accordance of his facts is distinctly shown on comparing those ascertained by Davy with those of Berthold, on the chelonian reptiles and the true amphibia; and also with those of Czermak and Welford, on the ophidian and saurian reptiles. Thus, even in the higher reptiles, the Chelonia, Davy found in one species, *Testudo Mydas*, a temperature of 84°, when that of the atmosphere was 79·5° Fahrenheit; and in another individual of this species, when the air was 80° Fahrenheit, a temperature of 88·5° Fahrenheit; and in a third which had been caught on the day previous, a temperature of only 85°, when that of the air was 86°. These differences, we have no doubt, arose, as in some of Hunter's experiments, from the individuals having been removed from a medium much colder than themselves, or from their being exposed to an atmosphere, the temperature of which was either rising or sinking rapidly, and in which the low amount of respiration in the animals was insufficient to change the temperature of their bodies so quickly as that of the surrounding air. Berthold's experiments clearly prove this to be the case. In the winter, when the animal was torpid, he found the temperature of *Testudo Græca* equal only to that of the atmosphere, which was about 56° Fahrenheit, but in summer when the animal was active, it had a temperature of 74·18° Fahrenheit, that of the air being 73·62° Fahrenheit. The same individual in winter, when aroused by being placed in a very gradually increased artificial heat, acquired in the same space of time a

* Medical and Philosophical Essays; London, 1740.
† Philosophical Transactions, 1775-78.
‡ Baumgærtner und Ettingshausen, Zeitschrift für Physick; 1824, vol. iii.
§ Annals of Philosophy, vol. ii.
|| Neue Versuche über die Temperatur der kaltblütigen Thiere; Götting. 1835.

much higher temperature than the air, or a vessel of water placed beside it, the temperature of the turtle being 95° Fahrenheit, and that of the water 83·7 Fahrenheit. Dr. Davy observed a similar difference in the same species when examined at different temperatures. At the Cape of Good Hope when the air was 61° Fahrenheit, that of the turtle was 62·5 Fahrenheit; and at Calumbo, when the air was 80, that of a larger individual of the same species was 87° Fahrenheit. In the true amphibia, a temperature lower than that of the surrounding medium is of very frequent occurrence. This may be attributed to the conjoint influence of two circumstances: first, a deficiency of power to generate heat so rapidly as it passes off from the body by means of evaporation, when the temperature of the surrounding medium is rising quickly; and next, and perhaps chiefly, to the much larger amount of heat, which is abstracted from the body in proportion to the amount of exhalation from the surface, which goes on more quickly in proportion to the increased temperature of the surrounding air.

On the temperature of fishes Dr. Davy has added some new and valuable facts. Previously to his observations it was supposed that the temperature of this class did not amount to more than one or at most two degrees above that of the medium in which the animals exist. Some authors even have denied entirely the existence of an independent temperature in fishes; among these are the distinguished naturalists, Humboldt and Provençal. But Dr. Davy has shown, in accordance with the statements of Martine, Hunter, Broussonet, and others, that not only do they generate an amount of heat above that of the surrounding medium, but that this amount varies in different species. In a shark just captured, and while still alive, when the temperature of the air was 71·75° Fahrenheit, and that of the sea 74·75° Fahrenheit, the temperature of the deep muscles of the back was 77° Fahrenheit. But the most remarkable instance of temperature in fishes is in the Bonito, in which, when the sea from which it was just taken was 80°, the deep muscles of the back had a temperature of 99° Fahrenheit; but the heat which is situated nearer the surface of the body was only 92° Fahrenheit: a difference not easily to be reconciled with Dr. Davy's experiments on some other animals.

On the heat of the Invertebrata, Dr. Davy's observations have fallen a little short of the facts. Although it is subject to vary under the influence of external circumstances, even to a greater extent than in most of the lower vertebrata, it amounts to a degree in some instances scarcely inferior to that of the more perfectly organized animals. We have proofs of this in the whole of the gregarious hymenoptera. The whole of the insect tribes have a temperature above that of the surrounding medium. But this is not shown in the experiments before us, some of which seem to indicate a lower temperature in some species than that of the atmosphere. This error of observation has doubtless arisen from inattention to the circumstance of the temperature of the air having risen during a state of quiescence or inactivity of the insect. Thus the temperature of *Blatta orientalis*, the common cockroach, is stated to have been only 75° Fahrenheit, when that of the air was 83° Fahrenheit. We are confirmed in our opinion of the inaccuracy of this observation, and of its dependence upon the circumstance alluded to, by another observation which Davy made on the same species, in which he found the temperature of the insect 75° Fahrenheit, when that of the atmosphere was only 74° Fahrenheit. That none of the air-breathing insects have a lower temperature than the medium they inhabit, when this has been subject to but slight variations, or even when this has been changing, and the insects at the same time have continued in a state of great activity, is sufficiently proved by the observations both of Berthold and Newport.* Berthold observed a temperature in the larvæ of caterpillars of the white cabbage butterfly, of from 1·5° to 2·5° Fahrenheit lower than that of the atmosphere, as had formerly been noticed by one of the oldest and most careful enquirers of this country, Martine; and also a high temperature in hymenoptera, and the enquiries of Newport have shown not only the constant development of an independent temperature in all insects in a state of activity; but also that the amount of this varies in the different tribes, being greatest in those which are most active and respire the greatest quantity of air. In the crustacea also, as well as in the mollusca, Dr. Davy has failed to observe an independent temperature, although there are good reasons for believing that it really does exist. Rudolphi observed a temperature of two or more degrees in the common crawfish, *astacus fluviatilis;* Hunter, Spallanzani,† and Gaspard,‡ have noticed it in the air-breathing mollusca; Pfeiffer § in the bivalves, and Hunter in the common leech.

We have dwelt more especially on these measures of temperature in different animals as given by Dr. Davy, and have compared them with the observations of others, because of their great value as a series, and because as such they have been adopted as a standard of reference by Dr. Edwards in his paper on Animal Heat; || and further, because of their immediate connexion with the enquiry concerning its origin.

In the paper on *animal heat,* Dr. Davy formerly gave the results of a few experiments on the very young infant, and expressed an opinion which is opposed to the more recent experiments of Dr. Edwards, but which he has not seen occasion to alter in this republication of his labours. He believes the temperature of the young animal to be higher than that of its parent. In illustration of this he states that the heat of the axilla of a child just born was 98·5° Fahrenheit; that at the expiration of twelve hours it was 99° Fahrenheit, and for three days continued at the same temperature; that in the instance of a weakly infant, an hour after birth, the temperature did not exceed 96° Fahrenheit, while on the following day it had risen to 98·5° Fahrenheit; and that in another infant it was 99° Fahrenheit. Dr. Edwards, on the contrary, believes that the temperature of the very young animal is in every instance lower than that of the parent, and that the human infant does not offer an exception to the rule. Dr. Davy's opinion has been advocated by several physiologists, and seems at first to be supported by the numerous experiments he has made on the temperature of children in temperate and tropical climates. But notwithstanding these the opinion of Dr. Edwards is substantially correct. The children experimented on by Davy, varied in age from

* Philosophical Trans., 1837. † Mém. sur la Réspiration.
‡ Majendie, Journal de Physiol, vol. ii.
§ Naturgeschichte deutscher Land und Süsswasser Mollusken; Weimer, 1825.
|| Cyclopædia of Practical and Comparative Anatomy, vol. ii.

four to fourteen years, a period in which, as Dr. Edwards has since shown,* the temperature of the child is indeed higher than that of the adult, while that of the very young infant is lower. He believes this difference to amount to so much as two degrees. We do not admit the existence of so great a difference as this, but we are satisfied of the fact, not only that the temperature of the child is lower than that of its parent, but also that the very young infant is incapable of maintaining the heat it is able to generate, when exposed to cold or removed from the warmth of its parent; and this opinion is further confirmed by the well-known fact, that the less mature the infant, the more constantly must heat be supplied to it from external sources, in order to assist its development.

But independently of the age of an animal, its temperature is now known to vary in different states of health. In a pathological point of view, therefore, the ascertainment of the real temperature of the body is a matter of great importance. In the insane, as Dr. Davy states, it has usually been supposed to be lower than in other individuals. It was desirable, therefore, to ascertain whether this opinion is correct, since the practitioner in his treatment of those labouring under this, the most humiliating and distressing of all infirmities, is often obliged to judge of the state of health almost entirely by the few symptoms which present themselves, the patient making no complaint of illness but, on the contrary, when questioned on the subject, pronouncing himself in good health, even when suffering from severe organic disease. Piorry† and others have shown that in fevers, and diseases of the lungs and other structures, the heat of the body may become raised to many degrees above the normal standard. The importance of attention to this subject is well remarked on by Dr. Davy. He believes that an attention to the temperature and pulse will often lead to the detection of disease not before suspected to exist, as was directly proved in a case described by him. The patient, who made no complaint, had a good appetite, and performed his functions naturally, on being found with an excited pulse, and a temperature under the tongue of 104·5° Fahrenheit, was more closely examined, and was then ascertained to be suffering from extensive disease with vomica, tubercles, and ulceration of the intestines and disease of the generative organs, but all unaccompanied by the usual symptoms.

Contrary to the generally received opinion, Dr. Davy found that the temperature of the insane certainly is not lower than that of other individuals. On examining the table which he has given of the temperature of twenty-four individuals, the mean age of whom, between the extremes of thirty-one and sixty-nine, was forty-seven years and about nine months, we find that it is not slightly, as stated by him, but considerably higher than that of other persons. Thus the mean of these twenty-four individuals, examined in winter, on the 17th of January, when the temperature of the atmosphere was 30° Fahrenheit, amounted to 99·16° Fahrenheit, the extremes being 98° and 102°; but in summer, on the 4th of August following, when the air was 68°, the mean of their temperature was 104·06° Fahrenheit, the extremes being 99° and 104·50°. These are valuable and interesting facts, and merit further enquiry.

* Cyclopædia of Practical Anatomy, art. Heat.

† Traité de Diagnostic et de Semeiologie, par P. A. Piorry. 1838.

Another pathological condition to which Dr. Davy has directed his attention, is *the effect of violent exercise on the temperature of the body* in a state of health. The observations are too few to enable us to deduce from them any positive conclusion, but are believed by him to show that during violent exercise the temperature becomes reduced. The observations, it should be remembered, were made in a hot climate, where the functions of the skin are more active than in milder regions.

The temperature of the human body after death is another subject of pathological interest, which we cannot omit to notice. In this paper Dr. Davy has directed attention to facts, which, when reduced to general principles, by means of a large number of carefully repeated observations on the rate of cooling of the body under different circumstances and in different temperatures and hygrometric states of the atmosphere, may hereafter become of great value in medico-legal enquiries. But a vast number of carefully repeated experiments are necessary before this important result can be hoped to be attained. Even when reduced to the precision of certain well-ascertained laws, its employment in difficult cases of medical jurisprudence, as Dr. Davy remarks, can only be truly useful in the hands of the most cautious and discerning. That it will ultimately become so, through the labours of many observers, we have not the slightest doubt, and we feel that much credit is due to him for commencing the enquiry. On examining the details now given we are not a little surprised at the remarkably high temperature that sometimes exists. Thus in one case of rheumatism, examined three hours and a half after death, when the temperature of the room was 86° Fahrenheit, after some of the viscera had been exposed for nearly ten minutes the mercury of a thermometer placed under the left ventricle rose to 113° Fahrenheit, and when in contact with the liver, the lobulus spigelii, to 112° Fahrenheit. In a second subject, examined six hours after death, the thermometer under the left ventricle indicated a temperature of 108° Fahrenheit, and when in contact with the lobulus spigelii 107° Fahrenheit. In these cases the patients were ill but a short time, and died suddenly, and Dr. Davy is decidedly of opinion that this high temperature had been acquired before death. It is interesting to observe that the bodies of those who died of slow fevers did not show so high a temperature. The highest was in those in which disease was most active. These circumstances strongly remind us of the high temperatures given by Piorry, and noticed in a former number of this Journal,* but which we were at that time disposed to receive with some hesitation. The subject merits more extensive examination, as well in regard to the temperature in disease before and after death.

*The experiments and observations on the blood* lead us to the consideration of the important changes which this fluid undergoes in the course of the circulation and when submitted to the process of respiration, as well as to the relation which they bear to the development of animal heat. We have seen in the above notices of Dr. Davy's papers the great difference of amount in the temperature of different animals, the variation it is subject to in different seasons, climates, periods of life, and states of health; and the questions naturally arise, what are the causes

* British and Foreign Med. Review, vol. VI. p. 142.

of this difference, and the circumstances attendant upon it? That the heat of the body is the result of certain changes in the blood is now, we believe, almost universally admitted; but it is still questioned by some whether, as most English physiologists believe, these changes, and the accompanying evolution of heat, are the necessary consequences of a chemical action of the air on the blood, or whether the evolution of heat results directly from the influence of the nervous system? Dr. Davy inclines to the opinion "that it is owing, first, to the fixation or condensation of oxygen in the blood in the lungs, in conversion from venous to arterial; and secondly, to the combinations into which it enters in the circulation in connexion with the different secretions and changes essential to animal life." (vol. ii. p. 171.) All the facts noticed in these experiments on the blood go to support this opinion; but we cannot afford space in this article, which has already exceeded the limits we had proposed for it, to examine them.

Dr. Davy's more recent experiments (the results of which differ considerably from those of his earlier ones, in consequence of his employing a more perfect apparatus) confirm those of Magnus, in regard to the presence of free gases in the blood, and the predominance of oxygen in arterial blood, and of carbonic acid in venous. They also show the lungs to be both absorbing and excreting organs.

All the facts noticed by Dr. Davy, and many others to which we could refer, tend to show that the evolution of heat is dependent chiefly on the chemical effect of the process of respiration, and also to explain the cause of the variation in the amount of heat in different animals, states, and periods of life. The highest temperature of body is in birds, and it is this class of animals which have the greatest rapidity of circulation and frequency of respiration, and which endure the privation of air with greater difficulty than any other animals. On the other hand it is in the lowest of the vertebrata, the reptiles and amphibia, which have a slow circulation, and can endure the privation of air for a long time, that there is the lowest temperature of body. Now these extreme conditions of bodily heat occur at stated periods in the same individuals of different tribes of mammalia, the hybernating animals. The hedgehog, the dormouse, and the bat, in their season of activity, greatly resemble birds in all their functions; the rapidity of circulation, frequency of the acts of respiration, and a high temperature of body; while like them they perish quickly when deprived of atmospheric air. But at another period, these same animals closely resemble the reptile in supporting, uninjured, for a long time, the absence of air, in the almost entire cessation of their circulatory and respiratory functions, and in the reduction of their temperature almost to that of the surrounding medium. Similar extreme conditions of life are observed in the same individuals in some of the insecta, both in regard to the activity at one season and to the infrequency at another of the respiratory and circulatory functions, and to a greatly augmented or a scarcely appreciable temperature. A striking instance of this variation in its vital functions is seen in the humble bee, which in its state of greatest activity has a temperature of body scarcely inferior to that of many mammalia, respires a very large proportion of atmospheric air, and perishes as quickly as other animals if deprived of it at that season; but at another time, like the true hybernating vertebrata, it remains for many weeks in a state of total inactivity, and requires for its support not one thousandth part of the air it deteriorates at another season, and evolves no perceptible heat.*

But notwithstanding the apparent strength of the above facts to support the opinion that animal heat is the direct result of changes induced in the blood through the agency of respiration, we cannot omit to notice that there are certain conditions of the body which at first seem opposed to this conclusion, and to lead to that which regards the evolution of heat as resulting from the direct agency of the nervous system. We have ourselves witnessed cases of paralysis of the whole of the voluntary muscles of the body, including some of those of respiration, in which there existed a very high temperature for many hours before death, and this too when the function of respiration was performed with much difficulty. But in each of these cases the activity of the circulatory organs had been greatly increased, so that a greater quantity of blood had passed through the vessels in a given time; a circumstance sufficient in itself, we think, to account for the increased evolution of heat, whatever might be assigned as the cause of this increased activity of the organs.

But our exhausted limits warn us to close this article. We feel assured that what we have said of Dr. Davy's labours will sufficiently convey the very high estimation in which we hold these volumes, and that it is needless for us again, in formal terms, to express the gratification we have derived from their perusal, or our conviction that they will conduce very much to the interest of science.

* Newport, Philosophical Trans. 1836-7.

